# Preperitoneal pelvic packing in patients with hemodynamic instability due to severe pelvic fracture: early experience in a Korean trauma center

**DOI:** 10.1186/s13049-016-0196-5

**Published:** 2016-01-13

**Authors:** Ji Young Jang, Hongjin Shim, Pil Young Jung, Seongyup Kim, Keum Seok Bae

**Affiliations:** Trauma Center, Department of Surgery, Wonju Severance Christian Hospital, Yonsei University Wonju College of Medicine, 220-701 Ilsan-ro, Wonju-si, Gangwon-do Republic of Korea

**Keywords:** Pelvis, Hemorrhage, Preperitoneal pelvic packing, Extraperitoneal pelvic packing, Damage control

## Abstract

**Background:**

The mortality rate of patients with hemodynamic instability due to severe pelvic fracture is reported to be 40–60 % despite a multidisciplinary treatment approach. Angioembolization and external fixation of the pelvis are the main procedures used to control bleeding in these patients. Several studies have shown that preperitoneal pelvic packing (PPP) is effective for hemorrhage control, despite being small and observational in nature. The purpose of this study was to describe a Korean trauma center’s early experience with PPP in unstable patients with pelvic fractures and to evaluate its effectiveness.

**Methods:**

Between January 2012 and May 2015, 30 patients with hemodynamic instability caused by pelvic fracture were enrolled in this study. PPP has been performed in 14 patients since May 2014. Data of pelvic fracture patients with hemodynamic instability were selected from Wonju Severance Christian Hospital Pelvic Trauma Database and were analyzed retrospectively.

**Results:**

Mean age and mean ISS were 60.4 ± 18.8 years and 39.2 ± 8.1 in 30 unstable patients with pelvic fracture. Mean SBP was 89.1 ± 24.7 mmHg, and mean hemoglobin was 10.6 ± 2.3 g/dL. When the non-PPP group (16 patients) and the PPP group (14 patients) were compared, there was no significant difference in the age, gender, ISS, and occurrence of associated injury (*p* = 0.82, *p* = 0.23, *p* = 0.92, and *p* = 0.60, respectively). Mortality rate due to acute hemorrhage were 37.5 % in the non-PPP group and 14.3 % in the PPP group. In the PPP group, three patients underwent PPP in the hybrid operating room, and a laparotomy was performed in three patients. Mean systolic blood pressure increased significantly after PPP (71.6 ± 9.8 vs. 132.2 ± 36.4 mmHg, *p* = 0.002).

**Conclusions:**

In unstable patients with pelvic fractures, PPP can be used as an effective treatment, complementary to AE, to control pelvic bleeding.

## Background

The mortality rate of patients with hemodynamic instability due to severe pelvic fracture is reported to be 40–60 %, despite a multidisciplinary treatment approach [1–6]. Trauma surgeons have a few treatment options, including angioembolization (AE) or external fixation (EF) of the pelvis [7]. However, arterial bleeding occurs only in about 10–15 % of cases; in the majority of cases, the hemorrhage is from an injured vein or fractured pelvic bone [8, 9]. In addition, AE is difficult to use in hemodynamically unstable patients, as it is a time-consuming procedure and patients have to be sent to an interventional suite. There are few other surgical treatment options if hemodynamic instability remains after AE. Several studies in Europe and the United States have investigated preperitoneal (retroperitoneal or extraperitoneal) pelvic packing (PPP) [10–14]. These studies showed that PPP was effective in hemorrhage control, although they were small in scale and observational in nature [10, 11, 13]. The purpose of this study was to describe a Korean trauma center’s early experience with PPP in patients with hemodynamic instability due to severe pelvic fracture, and to evaluate its effectiveness.

## Methods

### Patient selection

Between January 2012 and May 2015, 30 patients with hemodynamic instability caused by severe pelvic fracture were enrolled in this study, among 1,164 severe trauma patients (injury severity score (ISS) > 15) who were admitted to the trauma center of Wonju Severance Christian Hospital. This study was approved by the institutional review board (IRB No: CR315004-003). Medical data of pelvic fracture patients with hemodynamic instability were selected from Wonju Severance Christian Hospital Pelvic Trauma Database which was developed as a part of the Korean Trauma Data Bank, and were analyzed retrospectively. Hemodynamic instability was defined as persistent hypotension (systolic blood pressure < 90 mmHg) in spite of loading 2 L of crystalloid and transfusion of 2 units of packed red blood cells (RBCs). Electronic medical data were reviewed for patient demographics, injury mechanism, associated injuries, initial and postoperative hemodynamic status and laboratory findings, blood transfusion requirement, time to intervention, ISS, type of pelvic fracture (Young-Burgess classification), surgical complications, overall mortality and hemorrhage-induced mortality. The study exclusion criteria were as follows: diagnosed with a severe traumatic brain injury, defined as a head Abbreviated Injury Scale score of ≥ 4, and patients with cardiac arrest at the time of arrival to the emergency room.

### Patient management

Between January 2012 and April 2014, PPP was not applied and pelvic angiography (PA) was performed in cases where contrast extravasation was identified on abdomino-pelvic CT. From May 2014 onwards, PPP was adopted in these patients, and orthopedic surgeons decided whether to perform external fixation of the pelvis. For all of major trauma patients, an extended focused assessment sonography for trauma (e-FAST) was done to evaluate the occurrence of intra-thoracic or intra-abdominal hemorrhage.

Unstable pelvic fracture was defined as identification of pelvic ring complete disruption (iliosacral fracture or iliosacral fracture dislocation or symphysis pubis diastasis or sacral fracture) in anteroposterior (AP) radiograph of pelvis or abdomino-pelvic computed tomography (CT) scan. A pelvic binder was applied to reduce pelvic capacity in patients with an unstable pelvic fracture, except in the lateral compression types because there was possibility of additional injury. Pelvic binder was removed just before operation or pelvic angiography was started. In patients with pelvic EF, pelvic binders were not re-applied, and were applied just after operation or procedure in patients without EF. When patient became hemodynamically stable, pelvic binders were released, and trauma surgeon discussed with orthopedic surgeon about the timing of EF removal and the application of pelvic internal fixation. After initial transfusion was started with two units of O negative packed RBCs, cross matched packed RBC and fresh frozen plasma (FFP) were given as a 1:1 ratio according to massive transfusion protocol.

### Preperitoneal pelvic packing techniques

**Fig. 1:**
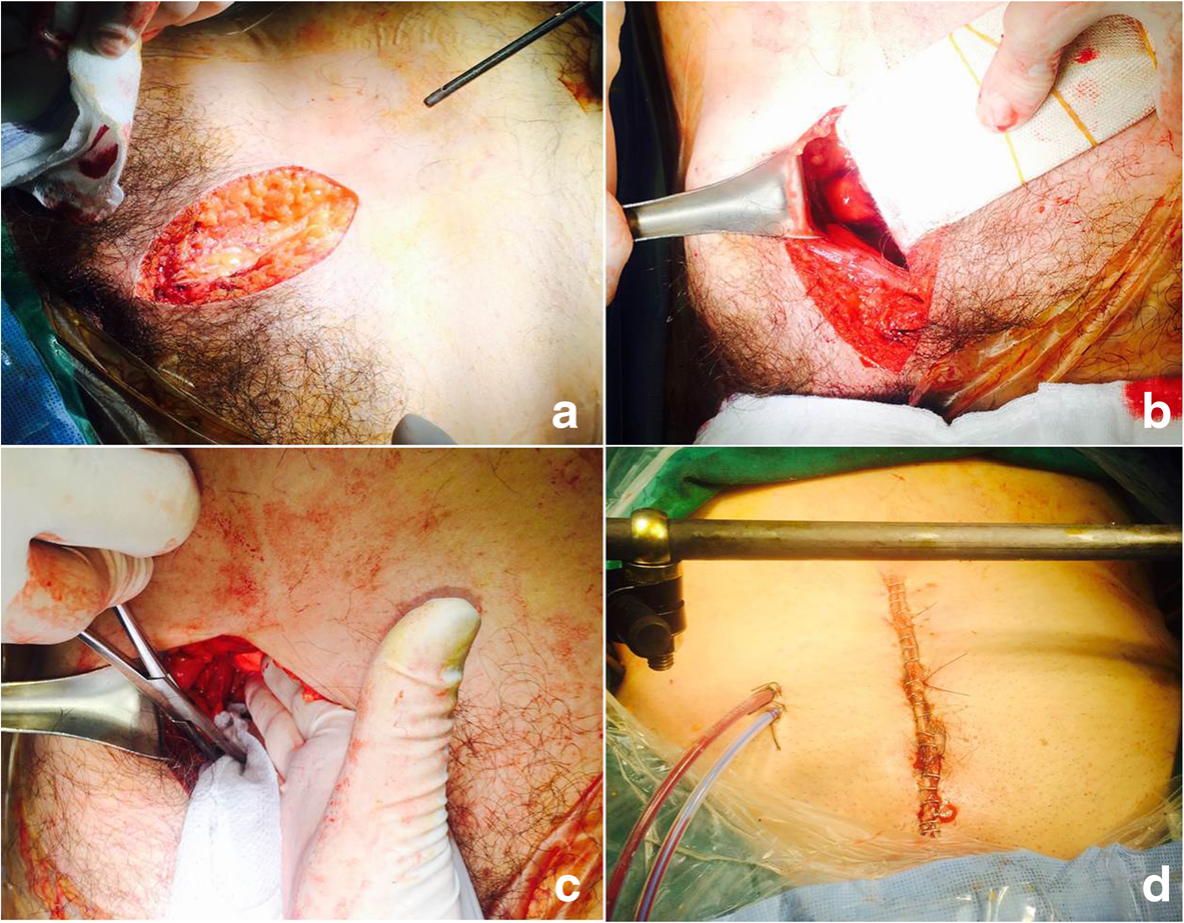
**a** Skin incision about 7–8 cm from the symphysis pubis. **b** Retraction of the peritoneum using a Deaver retractor. **c** Packing of surgical laparotomy pads. **d** Completion of the second operation

PPP was performed by trauma surgeons who completed the Definitive Surgical Trauma Care (DSTC) course provided by the International Association for Trauma Surgery and Intensive Care (IATSIC) [15]. During the procedure, the patient was placed supine and a 7–8-cm vertical skin incision was made starting at the symphysis pubis (Fig. 1a). After vertically resecting the anterior sheath of the rectus abdominis muscle and splitting the muscle, the peritoneum was palpated using a fingertip. Blunt dissection was performed through the preperitoneal space in the posterolateral direction to palpate the lateral border of the sacroiliac (SI) joint. Medial migration of the peritoneum with a Deaver retractor was used to improve the operative view where necessary (Fig. 1b). Three surgical laparotomy pads were then packed firmly from the near side of the SI joint using ringed forceps (Fig. 1c). The same procedure was repeated on the contralateral side and skin was approximated with a continuous suture. Then, external fixation was performed according to the orthopedic surgeon’s decision. After PPP, patients were sent to the trauma intensive care unit (TICU) and resuscitation and transfusion were maintained until patients stabilized. After the patient’s coagulopathy was sufficiently corrected, decisions regarding the need for a second operation were made, and if possible, it was performed within 48 h. During the second operation, the packed surgical laparotomy pads were removed and the bleeder was controlled. Then, a closed suction drain was inserted into the preperitoneal space and fascia repair was performed (Fig. 1d). When the amount of drainage decreased below 50 cc, the drain catheter was removed.

### Statistical analysis

All calculations were performed using SPSS version 19.0 (SPSS, Inc., Chicago, IL). Continuous variables are expressed as mean ± standard deviation or median (range). Differences between the two groups were compared by the chi-square, Fisher’s exact, Mann–Whitney U, and Wilcoxon signed rank tests. Statistical significance was accepted for p-values < 0.05.

## Results

### Comparison between the non-preperitoneal pelvic packing group and the preperitoneal pelvic packing group

Thirty patients were enrolled in this study. The mean age was 60.4 ± 18.8, and 22 patients (73.3 %) were male. The mean ISS was 39.2 ± 8.1, and 26 patients (86.7 %) had associated injuries with an AIS >2. Eleven patients (36.7 %) died, of which eight died from acute hemorrhage. When comparing the non-PPP group to the PPP group, there was no significant difference in the age, gender, ISS, or occurrence of associated injury (*p* = 0.82, *p* = 0.23, *p* = 0.92, and *p* = 0.60, respectively). The initial hemoglobin, initial lactate, transfusion requirements during the first four hours after admission and 24 h after TICU admission also showed no significant difference (*p* = 0.73, *p* = 0.92, *p* = 0.31, and *p* = 0.09, respectively). The mean time to emergency PA (194 ± 45 min) in the non-PPP group was significantly longer than the mean time to PPP (55.4 ± 28.6 min) in the PPP group (p < 0.00). Mortality rates due to acute hemorrhage were 37.5 % in the non-PPP group and 14.3 % in the PPP group, but this difference was not statistically significant (*p* = 0.226) (Table 1).

**Table 1:** Comparison between the non-PPP group and the PPP group

	Total (*N* = 30)	Non-PPP (*N* = 16)	PPP (*N* = 14)	*p*-value
Age (years)	60.4 ± 18.8	60.9 ± 22.1	59.7 ± 15.0	0.82
Male	22 (73.3 %)	10 (62.5 %)	12 (85.7 %)	0.23*
Injury severity score	39.2 ± 8.1	32.2 ± 4.9	38.8 ± 8.3	0.92
Associated injury (AIS >2)	26 (86.7 %)	13 (81.2 %)	13 (92.9 %)	0.60*
Systolic blood pressure on arrival (mmHg)	89.1 ± 24.7	84.3 ± 24.1	94.6 ± 25.1	0.29
Initial hemoglobin (g/dL)	10.6 ± 2.2	10.7 ± 2.5	10.4 ± 1.8	0.73
Initial lactate	5.0 ± 3.1	5.2 ± 3.4	4.9 ± 2.8	0.92
Red blood cell transfusion requirement over 4 h (units)	13.9 ± 10.9	11.3 ± 6.5	16.9 ± 14.2	0.31
Red blood cell transfusion requirement in the trauma intensive care unit (for 24 h)	5 (0–17)	1 (0–11)	7 (0–17)	0.09
Pelvic angiography	12 (40 %)	5 (31.3 %)	7 (50 %)	0.30
Time to emergency intervention (min)	132.2 ± 62.4	194 ± 45 (*n* = 4)	55 ± 27 (*n* = 10^a^)	<0.00
Overall mortality	11 (36.7 %)	6 (37.5 %)	5 (35.7 %)	0.92
Mortality due to hemorrhage	8 (26.7 %)	6 (37.5 %)	2 (14.3 %)	0.23*

PPP, preperitoneal pelvic packing

^a^ In three other cases, delayed PPP was excluded

* Fisher’s exact test

### Clinical characteristics of patients who underwent preperitoneal pelvic packing

In the PPP group, the most common injury mechanism was driver road traffic collision (RTC) (5, 35.7 %), followed by a fall from a significant height (3, 21.4 %), pedestrian RTC (3, 21.4 %), a crush injury (2, 14.3 %), and passenger RTC (1, 7.1 %). According to the Young-Burgess classification of pelvic fracture type, five patients (35.7 %) were found to have type 2 lateral compression, four (28.6 %) were found to have type 2 anterior-posterior compression, and two (14.3 %) were found to have vertical shear.

Six of seven patients underwent emergent PA and embolization was performed in two patients (33.3 %). One had arterial blush on initial abdomino-pelvic CT, and the other one underwent PA and embolization without CT scan prior to PPP, because of hemodynamic instability and delayed preparation of operative room. Laparotomy was performed concurrently in 3 patients, which included intra-peritoneal tape packing, splenectomy and segmental resection of the small bowel. The mean operation time for PPP with a concurrent operation was 46.6 ± 43.3 min, and the mean time for PPP alone was 29.7 ± 6.0 min. Among the seven patients (50 %) who underwent EF of the pelvis, six patients underwent EF immediately after PPP, and the remaining patient underwent EF the next day (Table 2).

**Table 2:** Clinical characteristics of patients who underwent preperitoneal pelvic packing (*N* = 14)

Variables	N (%)
Injury mechanism	
Road traffic collision	
Driver	5 (35.7)
Pedestrian	3 (21.4)
Crush	2 (14.3)
Passenger	1 (7.1)
Fall	3 (21.4)
Associated injury (AIS > 2)	
Head & neck	3 (21.4)
Face	3 (21.4)
Chest	9 (64.3)
Abdomen & pelvic contents	5 (35.7)
Pelvic fracture type (Young-Burgess type)	
Anterior posterior compression 2	4 (28.6)
Anterior posterior compression 3	1 (7.1)
Lateral compression 1	1 (7.1)
Lateral compression 2	5 (35.7)
Lateral compression 3	1 (7.1)
Vertical shear	2 (14.3)
Open pelvic fracture	2 (14.3)
Abdominopelvic CT/ arterial blush	11 (78.6 %)/1 (9.1 %)
Emergency pelvic angiography	6 (50)
Embolization	2/6 (33.3)
PPP time (min) (*n* = 10) ^a^	29.7 ± 6.0
Emergency external fixation	7 (50)
Hybrid operating room PPP	3 (21.4)

*AIS* abbreviated injury scale, *CT* computed tomography, *PPP* preperitoneal pelvic packing

^a^ Patients who underwent concurrent laparotomy and cystostomy were excluded

### Clinical outcomes of patients who underwent preperitoneal pelvic packing

In the PPP group, the mean time from admission to PPP was 55.4 ± 28.6 min in 10 patients, excluding three patients who underwent PPP after PA and one patient who got delayed PPP after TICU admission because of persistent hemodynamic instability. Two patients (66.7 %) died from acute hemorrhage among these three patients who underwent PA before PPP. Patients who underwent PA first had a higher mortality rate than patients who underwent PPP first (*p* = 0.03). The mean SBP after PPP was significantly higher than the mean lowest SBP before PPP (71.6 ± 9.8 vs 132.2 ± 36.4 mmHg, *p* = 0.002). In the PPP group, the median amount of packed RBCs and FFP units transfused in the ER were 5.5 (2–44) units and 4.5 (0–30) units, the mean amount of units transfused in the operating and intervention rooms were 11.7 ± 9.2 units and 6.6 ± 5.9 units, and the mean amount of units transfused in the first 24 h after TICU admission were 8.6 ± 5.5 units and 7.4 ± 4.7 units. The same number of platelet unit was transfused after TICU admission, because platelet had to be received from central blood bank. Among seven patients who underwent pelvic EF, open reduction and internal fixation (ORIF) for pelvic fracture were performed in 4 patients. Two patients could not undergo IF after pelvic EF, because of death. The other one underwent only pelvic EF. Among seven patients who pelvic EF was not applied, three patients underwent only ORIF for pelvic fracture. Neither EF nor ORIF was performed in four patients, two were due to early mortality, and the other two patients were managed conservatively by callus formation. Complications related with PPP occurred in four patients (28.6 %). Wound dehiscence occurred in two patients after second operation, it was possible to repair under local anesthesia. One patient was diagnosed with abdominal compartment syndrome after first operation, and was able to recover with conservative management. After surgical site infection was identified in one patient after third operation for pelvic internal fixation, he died from sepsis on hospital day 7. Six of nine survivors (66.7 %) were transferred to department of rehabilitation medicine after mean 40.5 ± 14.7 days from admission, and underwent rehabilitation therapy during about 60 days on average (65.7 ± 28.8 days). Five patients (35.7 %) died, of which two patients (14.3 %) died from acute hemorrhage (Table 3). When mean serum lactate in survivors and non-survivors whose mortalities were not caused by acute hemorrhage on TICU day 1,2 and 3, survivors had significantly lower mean serum lactate than non-survivors on TICU day 2 (survivor; 2.9 ± 1.8 vs non-survivor; 7.0 ± 2.8 mmol/L, *p* = 0.02) (Table 4).

**Table 3:** Clinical outcomes of patients who underwent preperitoneal pelvic packing (*N* = 14)

Variables	N (range)
Duration of ER stay (min)	107.8 ± 47.5
Time to PPP (min) (overall)	60.5 (15 – 501)
Time to emergent PPP (min) ^a^	55.4 ± 28.6
Time to emergency angiography (min)	77.8 ± 37.9
Lowest systolic blood pressure before PPP (mmHg)	71.6 ± 9.8
Lowest hemoglobin before PPP (g/dL)	9.4 ± 1.9
Lactate before PPP	4.9 ± 2.8
Systolic blood pressure after PPP (mmHg)	132.2 ± 36.4
Hemoglobin after PPP (g/dL)	10.0 ± 1.8
Lactate after PPP	7.3 ± 3.6
Red blood cell transfusion in the ER (units)	5.5 (2–44)
Fresh frozen plasma transfusion in the ER (units)	4.5 (0–30)
Red blood cell transfusion requirement before trauma intensive care unit admission (units)	11.7 ± 9.2
Fresh frozen plasma transfusion requirement before trauma intensive care unit admission (units)	6.6 ± 5.9
Red blood cell transfusion requirement in the trauma intensive care unit (units/ 24 hours)	8.6 ± 5.5
Fresh frozen plasma transfusion requirement in the trauma intensive care unit (units/24 hours)	7.4 ± 4.7
Time from PPP to tape removal (hours)	60.8 ± 20.9
Duration of mechanical ventilation	9.4 ± 5.8
Duration of trauma intensive care unit stay (days)	14.0 ± 9.4
Mortality (d/t acute hemorrhage)	2 (14.3 %)
All mortality	5 (35.7 %)

*ER* emergency room, *PPP* preperitoneal pelvic packing

^a^ Ten patients who underwent PPP as emergency operation

**Table 4:** Difference of serum lactate change during between survivor and non-survivor

Serum lactate level (mmol/L)	Survivor (*n* = 11)	Non-survivor (*n* = 3) ^a^	*p*-value
Trauma intensive care unit day 1	3.2 ± 1.4	3.6 ± 0.5	0.63
Trauma intensive care unit day 2	2.9 ± 1.8	7.0 ± 2.8	0.02
Trauma intensive care unit day 3	1.9 ± 1.2	6.4 ± 4.5	0.22
Delta value of serum lactate (Day 1 – Day 3)	−1.4 ± 1.3	2.8 ± 4.8	0.28

^a^Two mortality cases due to acute hemorrhage were excluded

Two out of five patients who died underwent PA prior to PPP. Among the others, 2 patients died from adult respiratory distress syndrome (ARDS), and 1 from septic shock caused by surgical site infection. One patient could not be sent to the OR due to severe hemodynamic instability, and therefore underwent PPP in the ER and a delayed pelvic AE was performed on postoperative day 2. Three patients underwent PA after PPP in the hybrid OR without transport to the interventional suite. In three of seven patients (42.9 %) who underwent PA, embolization was performed.

## Discussion

Because the majority of pelvic fractures are accompanied by injuries at other sites, it is often difficult to determine whether concurrent hemodynamic instability originates from the pelvic cavity or from another injury site. Therefore, a management algorithm when there is the possibility of pelvic fracture is essential in the initial diagnosis of blunt trauma. Although e-FAST has facilitated the rapid detection of chest or abdominal injury, it is often ineffective in identifying hemorrhage of the pelvic cavity in early resuscitation. In cases where shock occurs in the initial phase, intra-thoracic or intra-abdominal hemorrhage is ruled out, and pelvic fracture is identified, physicians have to suspect hemorrhage due to severe pelvic fracture and start aggressive management. In our hospital, a new pelvic trauma management algorithm including PPP was developed and has been applied to patients since May 2014.

The present study showed that there was no difference in ISS and initial hemodynamic parameters except for the occurrence of PPP application between the non-PPP group and the PPP group. There was no difference in overall mortality rate between both groups (37.5 % vs. 35.7 %), however there was a difference noted in the mortality rate due to acute hemorrhage, which were 37.5 and 14.3 %, respectively. While all six mortality cases in the non-PPP group were caused by acute hemorrhage in the ER, only two (40 %) of the mortality cases were caused by acute hemorrhage in the PPP group. In other words, it appears that acute hemorrhage in the PPP group was successfully controlled and these patients could be sent to the TICU. In addition, we found that the time to PPP in the PPP group was significantly shorter than the time to PA in the non-PPP group. These results are similar to those of a previous study that compared patients who underwent early PA to patients who underwent early PPP [14]. It seems that the longer time required to PA was caused by the absence of on-site angiography personnel. Considering that the mean time to PPP was 29.7 ± 6.0 min, PPP has an advantage in reducing the time to hemorrhage control, because PPP can be performed quickly and is an easy procedure. Two of 14 deceased patients in the PPP group underwent PA prior to PPP (*p* = 0.03) and embolization was needed in only two (33.3 %) of six patients who underwent AE, which is also similar to the results of previous studies where the main bleeding source in pelvic fracture was fractured bone or venous plexus injury [8, 9]. In addition, transport of unstable patients to an interventional suite is hazardous, and time will be consumed in cases without arterial hemorrhage. The Denver Trauma Center in the US reported that there was no mortality due to acute hemorrhage in 75 unstable pelvic fracture patients with application of PPP/EF with secondary AE protocol [13]. Taken collectively, we suggest that PPP be performed prior to PA if advantageous.

Recently, a hybrid suite or RAPTOR (Resuscitation with Angiography, Percutaneous Techniques and Operative Repair) unit has been introduced, which aids in performing percutaneous procedures, interventional and diagnostic radiology, open operations and resuscitation in a single space. [16, 17]. The present study included three cases in which PPP and PA were performed in the hybrid OR. From November 2014 onwards, unstable pelvic fracture patients were sent to the hybrid OR in our hospital after initial resuscitation, PPP was performed first, and then if shock status persisted, PA was done in the same OR. This helped reduce the time to adequate hemorrhage control by eliminating transport from the OR to the intervention suite, and additional injuries were avoided by diminishing unnecessary movement. An anesthesiologist was able to perform hemodynamic monitoring and airway management during these procedures.

In South Korea, a national program to establish regional trauma centers was developed in 2012. A trauma center was established in 2012 in our hospital with a multidisciplinary trauma team consisting of general surgeons, cardiothoracic surgeons, neurosurgeons, anesthesiologists, interventional radiologists, and emergency medicine doctors. Because personal firearms are prohibited in Korea, blunt trauma accounts for about 90 % of all trauma cases [18]. In the management of unstable pelvic fracture, which has a high mortality rate, a multi-disciplinary team approach is crucial [19]. Because effective pre-hospital transport systems have not been completed in Korea, transport of pelvic fracture patients is often delayed. In addition, because of a lack of interventional radiologists, it often takes a long time to perform PA after admission. Given this situation, it seems that PPP is a useful procedure to initially control hemorrhage in pelvic fracture patients.

The major limitations of our study are that the sample size may be too small to confirm the effectiveness of PPP and that the data was analyzed retrospectively. During the early PPP period, there was no definitive protocol regarding the sequence of PPP and PA. In spite of these limitations, this study shows that PPP can be used successfully with PA in pelvic fracture patients with hemodynamic instability, and provides meaningful information about the early experience with PPP in a South Korean trauma center. Future prospective and large-scale studies on this topic are needed.

## Conclusion

In patients with hemodynamic instability due to severe pelvic fracture, PPP can be used as an effective treatment choice, complementary to PA, in order to control pelvic bleeding.
